# Digital transformation as a driver of operational sustainability in manufacturing: a systematic review (2015–2025)

**DOI:** 10.12688/f1000research.178740.2

**Published:** 2026-09-16

**Authors:** Luis Edgardo Cruz Salinas, Jessica Macalopú Rimachi, Carlos José Sandoval Reyes

**Affiliations:** 1 La Libertad, Universidad Cesar Vallejo, Chepén, La Libertad, 13871, Peru

**Keywords:** digital transformation, operational sustainability, manufacturing industry, digital twins, Industry 4.0, systematic review

## Abstract

**Objective:**

This research examines how the adoption of digital technologies—the Internet of Things (IoT), artificial intelligence, digital twins, and big data analytics—affects operational sustainability in the manufacturing sector.

**Design/methodology:**

A systematic review was conducted in accordance with the PRISMA 2020 guidelines, consulting Scopus, ScienceDirect and Taylor & Francis for the period 2015–2025. Of 1,247 initial records, 50 studies passed the eligibility and methodological quality filters.

**Findings:**

The evidence indicates that the joint implementation of several digital technologies produces reductions of between 20% and 35% in energy consumption and between 25% and 40% in material waste, according to the ranges reported in the primary studies. Digital twins had the greatest effects on process optimisation, followed by industrial IoT and artificial intelligence systems. Organisations that combined three or more complementary technologies achieved improvements 40%–60% greater than isolated implementations.

**Originality:**

The work offers a structured synthesis of the specific mechanisms that connect digitalisation with concrete sustainability indicators and identifies knowledge gaps that guide future lines of research.

## I. Introduction

The manufacturing sector today operates under dual pressure: to remain competitive in global markets while reducing its environmental footprint ( Chiarini and Kumar, 2021; Luthra et al., 2020). This sector generates approximately 16% of global GDP and employs hundreds of millions of workers, so any advances in its sustainability practices have far-reaching economic and social repercussions ( Xi et al., 2024; Ahmadi-Gh and Bello-Pintado, 2024).

Technologies associated with Industry 4.0—industrial IoT, artificial intelligence, digital twins, big data analytics, and cyber-physical systems—have been proposed as the strategic response to this dual challenge ( Attaran et al., 2024; Dong et al., 2025). In theory, these tools make it possible to monitor resource consumption in real time, anticipate failures using predictive models, and optimise production configurations without shutting down the plant. However, the literature shows that effective adoption remains uneven and that the links between digitalisation and operational sustainability are not fully documented ( De Felice et al., 2024; Pigola et al., 2025; Kiel et al., 2017).

This research revolves around the question: how does digital transformation contribute to the operational sustainability of the manufacturing industry during the period 2015–2025? To answer this question, a systematic review of scientific literature was designed that: (a) identifies the digital technologies with the greatest impact on operational sustainability indicators; (b) compares the effectiveness of integrated approaches versus isolated implementations; and (c) detects knowledge gaps that limit the generalisation of results.

Interest in this intersection has grown rapidly. Luthra et al. (2020) identified Industry 4.0 as a catalyst for sustainable practices in the supply chains of emerging economies. Subsequently, Chiarini and Kumar (2021) showed that combining Lean Six Sigma with Industry 4.0 tools led to measurable reductions in waste and energy consumption in Italian companies. Dong et al. (2025) documented that Chinese companies with broad digital adoption developed more green innovation capabilities than those with partial implementations. More recently, Singh et al. (2024) applied grey influence analysis (GINA) to assess the role of digital twins in the resilience of manufacturing supply chains.

However, significant gaps remain. There are few longitudinal studies documenting the evolution of benefits over time. Most of the evidence comes from Europe and Asia-Pacific, with little representation from Latin America and Africa. Furthermore, there is a lack of standardised evaluation frameworks that allow for the comparison of results across different sectors and geographical contexts. Taken together, these shortcomings constitute a concrete scientific gap: the absence of a synthesis that (i) links specific digital technologies to specific operational sustainability outcomes, (ii) contrasts the effectiveness of integrated versus isolated technology adoption, and (iii) makes this evidence base transparent enough to be extended over time. This review aims to close that gap through a synthesis that integrates findings from 50 empirical and theoretical studies selected using strict quality criteria.

## II. Theoretical framework

Compared with earlier reviews of digitalisation and sustainable manufacturing (e.g., Sartal et al., 2020; Sharma et al., 2021), the novelty of the present review is threefold: it covers the most recent decade of evidence (2015–2025), including post-pandemic studies not captured by earlier syntheses; it focuses specifically on operational sustainability indicators (energy, waste, emissions, and cost) rather than sustainability in general; and it explicitly contrasts the performance of integrated multi-technology adoption against isolated single-technology implementations, a distinction that has not been systematically quantified in prior reviews.

The 2015–2025 timeframe was selected because it brackets the period in which Industry 4.0 technologies moved from early conceptualisation ( Stock and Seliger, 2016) to mainstream industrial deployment, providing sufficient volume and maturity of empirical studies to support a meaningful synthesis while remaining recent enough to reflect current technology generations.

### Global context of sustainable manufacturing

The convergence of environmental regulation, consumer expectations and climate goals has placed manufacturing at the centre of decarbonisation efforts ( Ghobakhloo et al., 2025; Monroy-Osorio, 2024). The 2030 Agenda and, in particular, SDGs 9 (industry, innovation and infrastructure) and 12 (responsible production and consumption) provide the regulatory framework that guides these transformations. In practice, manufacturing companies must reconcile the pressure to reduce costs with the need to invest in less polluting processes ( Bag et al., 2023; Strandhagen et al., 2022; Sharma et al., 2021; Sartal et al., 2020).

The available evidence suggests that organisations that manage to align their digitalisation strategy with environmental objectives not only preserve their competitiveness but also develop advantages that are difficult to imitate, in line with the theory of dynamic capabilities ( Ahmadi-Gh and Bello-Pintado, 2024; Zekhnini et al., 2022; Hermawan et al., 2024). However, this assertion is still based on cross-sectional studies; causal confirmation requires longitudinal designs that are not yet sufficiently available in the literature.

### Fundamentals of digital transformation in manufacturing

Digital transformation is not limited to the ad hoc adoption of devices or software. As characterised by Paul et al. (2024), Attaran et al. (2024) and Ghobakhloo et al. (2025), it is a redesign of processes, structures and business models that leverages connectivity, automation and data intelligence. The main technological pillars include:

Industrial IoT enables the connection of machines, sensors and control systems to collect real-time operational data. Artificial intelligence and machine learning transform this data into energy consumption predictions, early fault detection and optimal production planning ( Monroy-Osorio, 2024; Rosa et al., 2020). Digital twins—virtual replicas of physical systems—enable the simulation of alternative production scenarios without stopping operations, facilitating experimentation with more efficient configurations ( Singh et al., 2024; Atalay et al., 2022). For its part, big data analytics extracts patterns of inefficiency that would remain invisible with conventional methods.

Núñez-Merino et al. (2020) and Chiarini et al. (2020) showed that these technologies function as enablers of lean principles: by providing total visibility of the process, they allow waste to be eliminated with greater precision. Karadayi-Usta (2024) extended this idea to the field of additive manufacturing, where the digitisation of the supply chain is a prerequisite for effective waste reduction.

### Operational sustainability: a multidimensional construct

In this paper, operational sustainability is defined as the ability of a manufacturing organisation to maintain increasing levels of production while simultaneously minimising the use of natural resources, waste generation and emissions, without compromising economic viability or the well-being of surrounding communities ( Pigola et al., 2025; De Felice et al., 2024; Chen et al., 2024).

This conceptualisation integrates the three dimensions of the Triple Bottom Line—economic, environmental, and social—and translates them into the realm of day-to-day operations. Mishra et al. (2024) developed and validated a scale that connects different dimensions of digitalisation with specific sustainability outcomes in the value chain. Their work provided quantitative evidence that digital traceability throughout the supply chain is positively associated with reductions in emissions and waste.

### Integration between digitalisation and sustainability: accumulated evidence

Between 2015 and 2025, the literature has shifted from linear models—where technology was conceived as an input for efficiency—to systemic frameworks that recognise interdependencies between technologies, organisational capabilities, and institutional context ( Xi et al., 2024; Pigola et al., 2025; Stock and Seliger, 2016).

Chiarini and Kumar (2021) documented in Italian companies that combining Lean Six Sigma with digital tools produced greater reductions in waste and energy consumption than those obtained by each approach separately. Dong et al. (2025) found, in a sample of Chinese companies, that the breadth of digitalisation had a greater impact on environmental resilience than the depth of implementation of a single technology. Strandhagen et al. (2022) applied this logic to the shipbuilding sector and confirmed that digitalisation addressed sustainability challenges specific to resource-intensive industries. Bag et al. (2023) further identified that digital technologies not only improve efficiency but also generate new capabilities for reuse and recycling ( Zaid et al., 2025).

The dynamic capabilities model ( Xi et al., 2024) and open innovation frameworks ( Singh et al., 2024; Maldonado-Guzmán and Pinzón-Castro, 2023; Bokrantz et al., 2020) offer a plausible explanation: companies that combine technological investment with internal skills development and external collaboration generate self-reinforcing sustainability advantages. However, this theoretical explanation needs further mpirical validation.

### Connection with the Sustainable Development Goals

Digital technologies contribute to SDG 9 by facilitating efficient, resilient industrial infrastructures with a lower carbon footprint ( Ghobakhloo et al., 2025). With regard to SDG 12, tools such as digital twins and blockchain-based traceability make it possible to monitor the environmental impact of a product throughout its life cycle, which promotes more informed production and consumption decisions ( Karadayi-Usta, 2024; Monroy-Osorio, 2024). Although the literature suggests that digitalisation can simultaneously drive several SDGs, it should be noted that the realisation of this potential depends on the strategic design of each implementation and on contextual factors that vary between regions and sectors.

## III. Methodology

### Study design

A systematic literature review was conducted following the PRISMA 2020 guidelines ( Page et al., 2021). The objective was to synthesise the available evidence on the relationship between the implementation of digital technologies and operational sustainability in manufacturing during the period 2015–2025.

### Search strategy

The search was conducted in three databases: Scopus, ScienceDirect and Taylor & Francis Online, selected for their coverage of high-impact journals in engineering, management and sustainability. The following combination of Boolean operators was used:

(“digital transformation” OR “digitalisation” OR “Industry 4.0” OR “digital technologies”) AND (“sustainability” OR “sustainable manufacturing” OR “operational sustainability” OR “environmental performance”) AND (“manufacturing” OR “manufacturing industry” OR “production systems”) AND (“operational efficiency” OR “operational performance” OR “process optimisation”)

Filters were applied for period (2015–2025), language (English and Spanish) and document type (peer-reviewed journal articles). Conference proceedings, books, theses and grey literature were excluded ( Sagala and Őri, 2024).

Searches were run against the title, abstract and keyword fields of each database. The Boolean string above was adapted to the specific search syntax of each platform (for example, adjusting phrase-quoting and proximity operators as required by Scopus, ScienceDirect and Taylor & Francis Online) while preserving the same underlying concepts and logic.

### Inclusion and exclusion criteria

Table 1 presents the inclusion and exclusion criteria applied in this review. Articles were included if they focused on digital technologies within the scope of Industry 4.0 and their effects on operational sustainability in manufacturing, were published in peer-reviewed journals between 2015 and 2025, and were written in English or Spanish.

**Table 1.** Inclusion and exclusion criteria.

Criterion	Inclusion	Exclusion
**Temporality**	Published between 2015 and 2025	Outside the range or speculative projections
**Theme**	Explicit relationship between digitalisation and sustainability in manufacturing	Digitalisation or sustainability addressed in isolation
**Type of publication**	Articles in peer-reviewed indexed journals	Technical reports, white papers, conference proceedings
**Sector**	Manufacturing industry or proven applicability	Exclusively service, agriculture or construction sectors
**Access**	Full text available	Restricted access preventing full evaluation
**Quality**	Rigorous methodology and transparent reporting	Serious methodological shortcomings

These criteria were established to ensure that the review captured empirically grounded, quality-controlled evidence directly relevant to manufacturing operations: the temporal window matches the diffusion period of Industry 4.0 technologies discussed in the Introduction; the requirement for an explicit digitalisation–sustainability relationship excludes studies that would not support associative or causal claims about this specific link; the restriction to peer-reviewed journal articles follows standard systematic-review practice for ensuring a minimum level of methodological scrutiny ( Page et al., 2021); and the language filter reflects the working languages of the review team while still covering the two languages in which most of the identified literature was published.

### Methodological quality assessment

Each study was independently evaluated by two reviewers using a checklist adapted from Downs and Black (1998) for quantitative studies and Tracy (2010) for qualitative studies. The criteria evaluated included: clarity in the definition of constructs, validity of instruments, adequacy of sample size, transparency in data reporting, and control of confounding variables. A scale of 0 to 10 was used, with scores of 7 or above classified as high quality, between 5 and 6 as moderate quality, and below 5 as low quality. Discrepancies were resolved by consensus between reviewers or, when necessary, with the intervention of a third evaluator.

To support transparency and reproducibility, the study-level quality scores generated with this instrument for all 50 included studies are reported in full in Extended Data Table 1.

### Selection process: PRISMA flow

**Figure 1.**
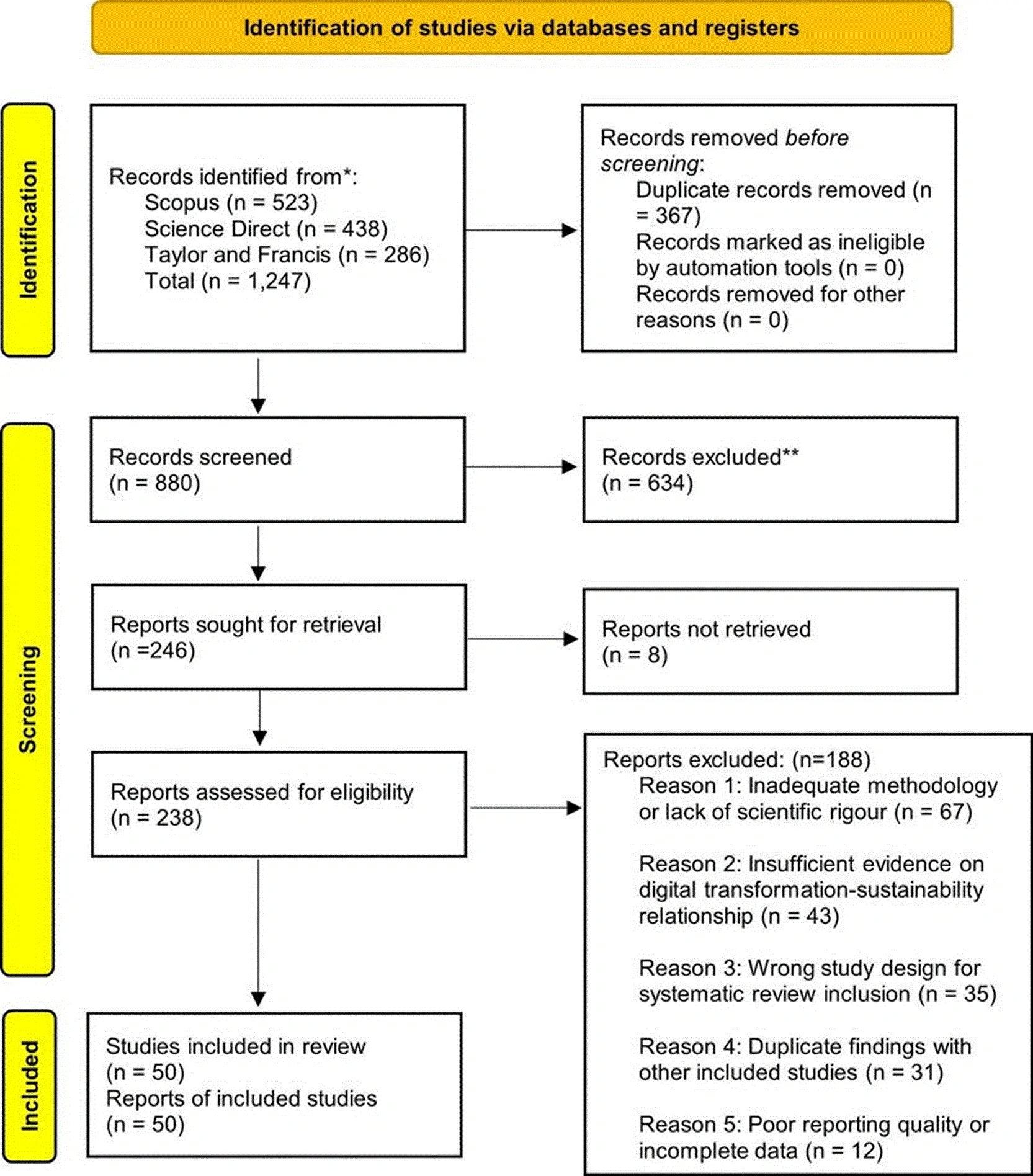
PRISMA 2020 flow diagram of the study selection process. The diagram illustrates the identification, screening, and inclusion stages, showing the number of records at each stage and the reasons for exclusion. A total of 1,247 records were identified; after removing duplicates and applying eligibility criteria, 50 studies were included in the final synthesis.

Figure 1 presents the flow diagram according to the PRISMA 2020 model. The initial search yielded 1,247 records. After removing 367 duplicates, 880 titles and abstracts were screened, of which 634 were discarded for not meeting the inclusion criteria. A total of 246 full texts were retrieved (8 were inaccessible), and of the 238 evaluated, 188 were excluded for the reasons detailed in the figure. The final sample consisted of 50 studies.

### Synthesis of results

Given the heterogeneity of designs (quantitative, qualitative, and mixed studies), a structured narrative synthesis complemented by thematic analysis was chosen. The information was organised into three areas: (a) digital technologies implemented and their mechanisms of influence; (b) sustainability indicators affected; and (c) moderating contextual factors. A standardised data extraction form was completed for each study.

Because a formal meta-analysis was not conducted, all percentage ranges and comparative figures reported in the Results and Discussion sections correspond to the ranges and point estimates as reported in the primary studies, recorded on the data extraction form; they should therefore be read as descriptive summaries of the reported evidence rather than as pooled or weighted effect-size estimates.

## IV. Results

### Profile of the studies included

**Figure 2.**
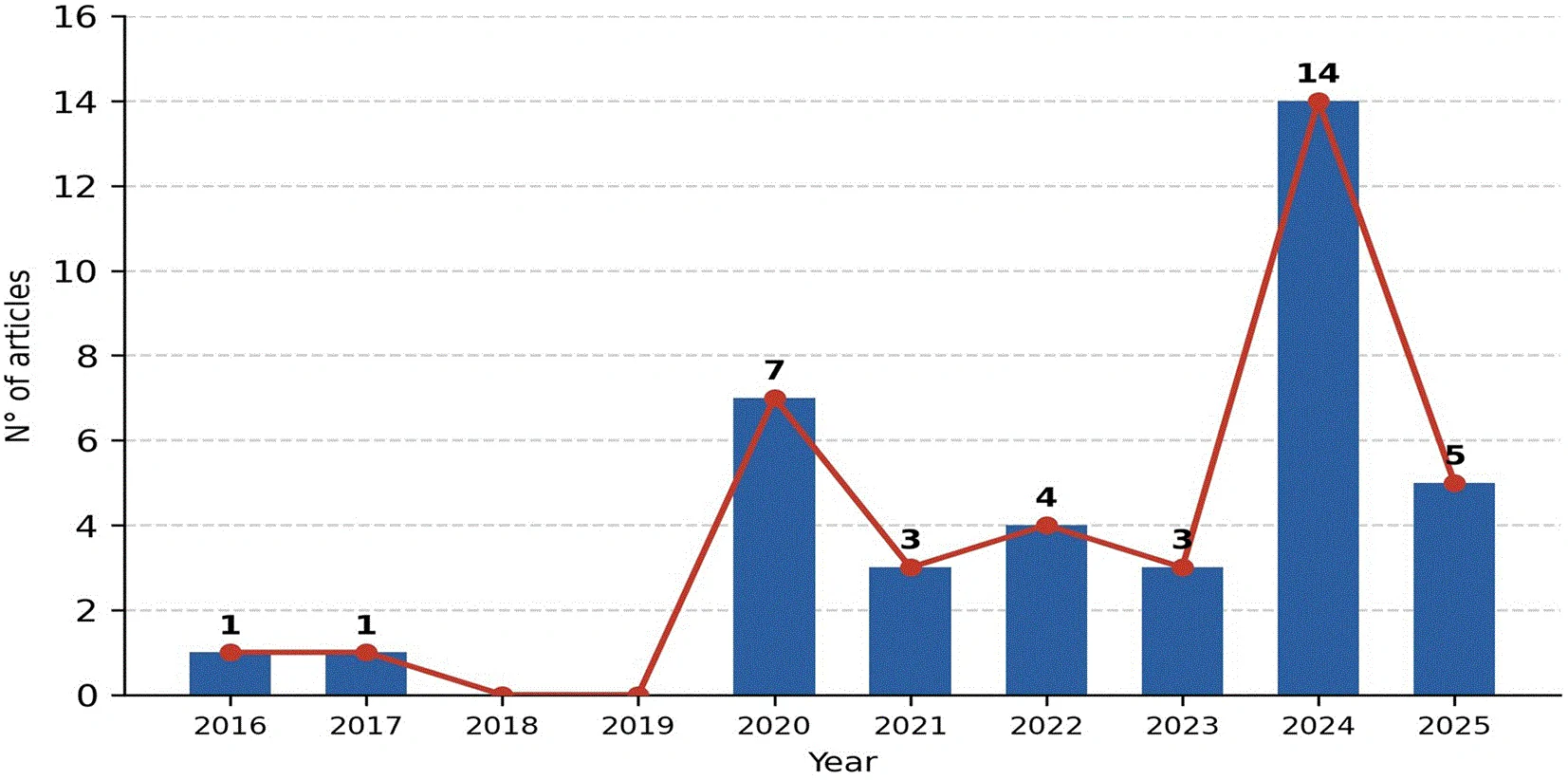
Annual scientific output of the studies included in the review (2015–2025). The bar chart shows the number of studies published per year, illustrating the rapid growth of research at the intersection of digital transformation and operational sustainability in manufacturing, particularly from 2020 onwards.

**Figure 3.**
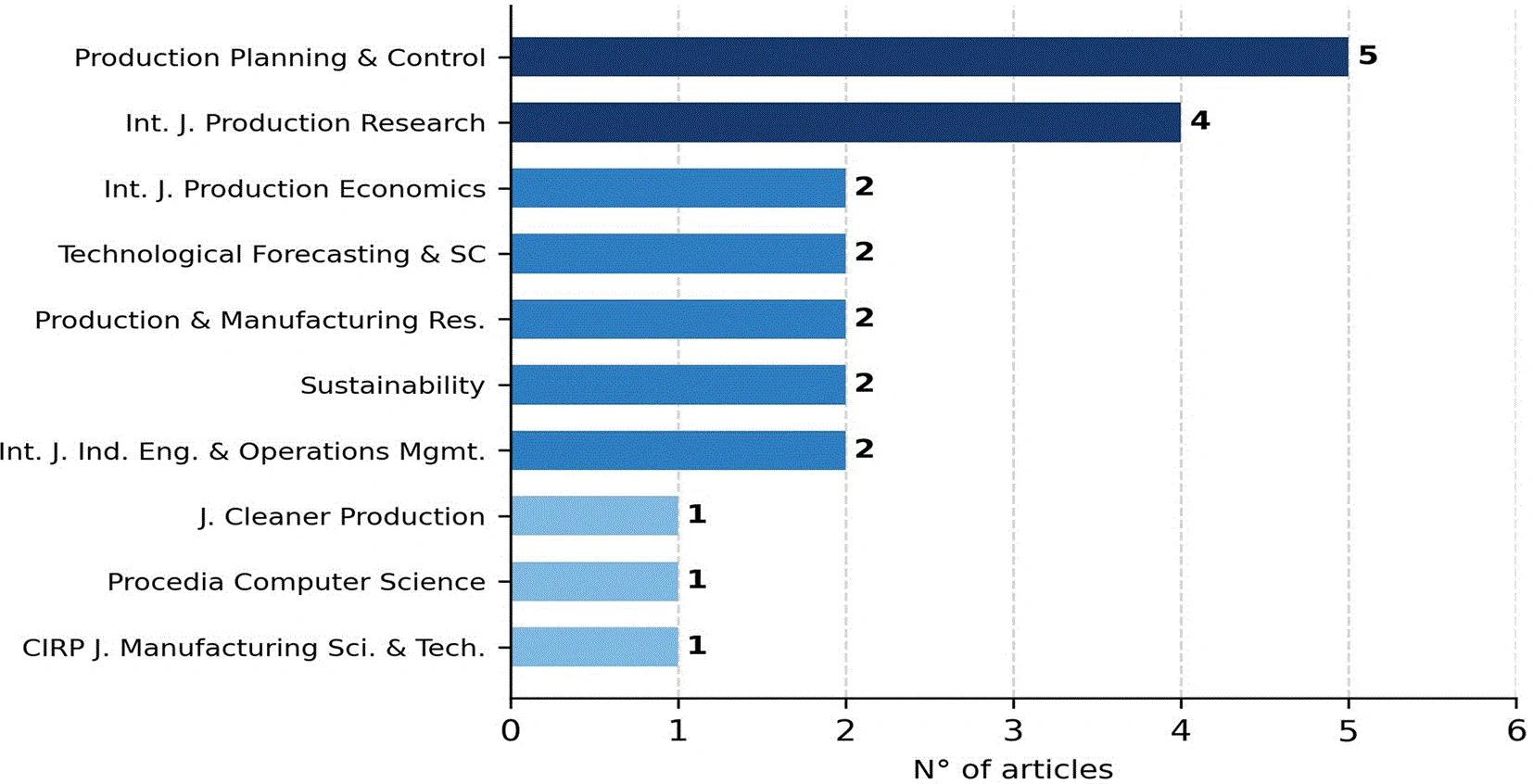
Distribution of studies by journal of publication. The chart shows the journals that contributed the most studies to this review, highlighting the interdisciplinary nature of the field across production engineering, sustainability, and management journals.

**Figure 4.**
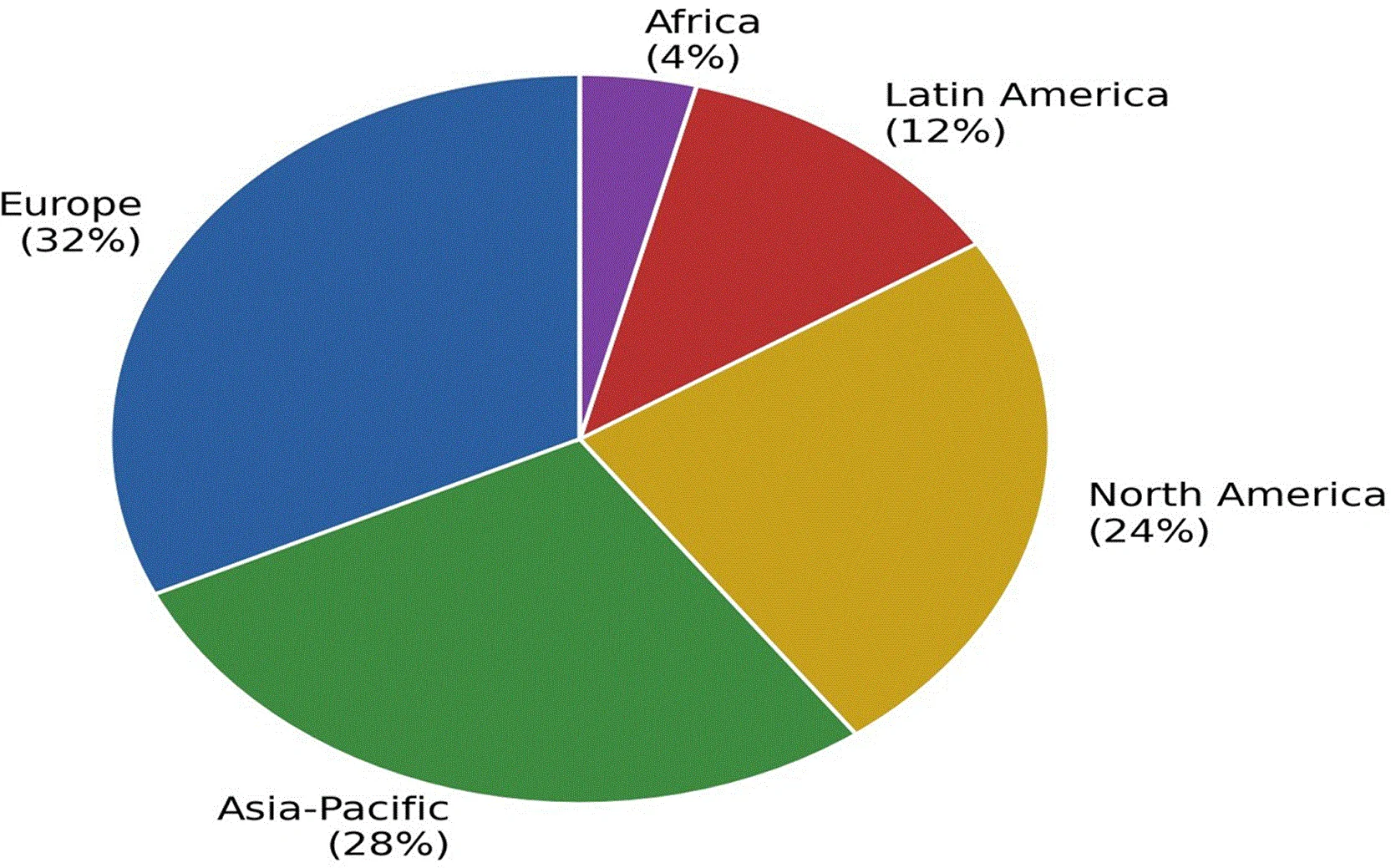
Geographical distribution of the studies included in the review. The map illustrates the uneven representation across regions, with Europe (32%), Asia-Pacific (28%), and North America (24%) accounting for the majority of studies, and Latin America (12%) and Africa (4%) underrepresented.

The 50 studies cover a variety of sectors: automotive (28%), electronics (22%), food (18%), textiles (14%), chemicals and pharmaceuticals (12%) and others (6%). In terms of the size of the organisations studied, 44% analysed large multinationals, 38% focused on SMEs, and 18% used mixed samples. The geographical distribution reflects differences in adoption maturity: Europe accounts for 32% of the studies, Asia-Pacific 28%, North America 24%, Latin America 12% and Africa 4% ( Yildiztekin et al., 2023; Song et al., 2022). Figure 2 shows the evolution of publications over time, Figure 3 presents the distribution by journal of publication, and Figure 4 presents the geographical distribution.

### Digital technologies evaluated and their effectiveness

**Figure 5.**
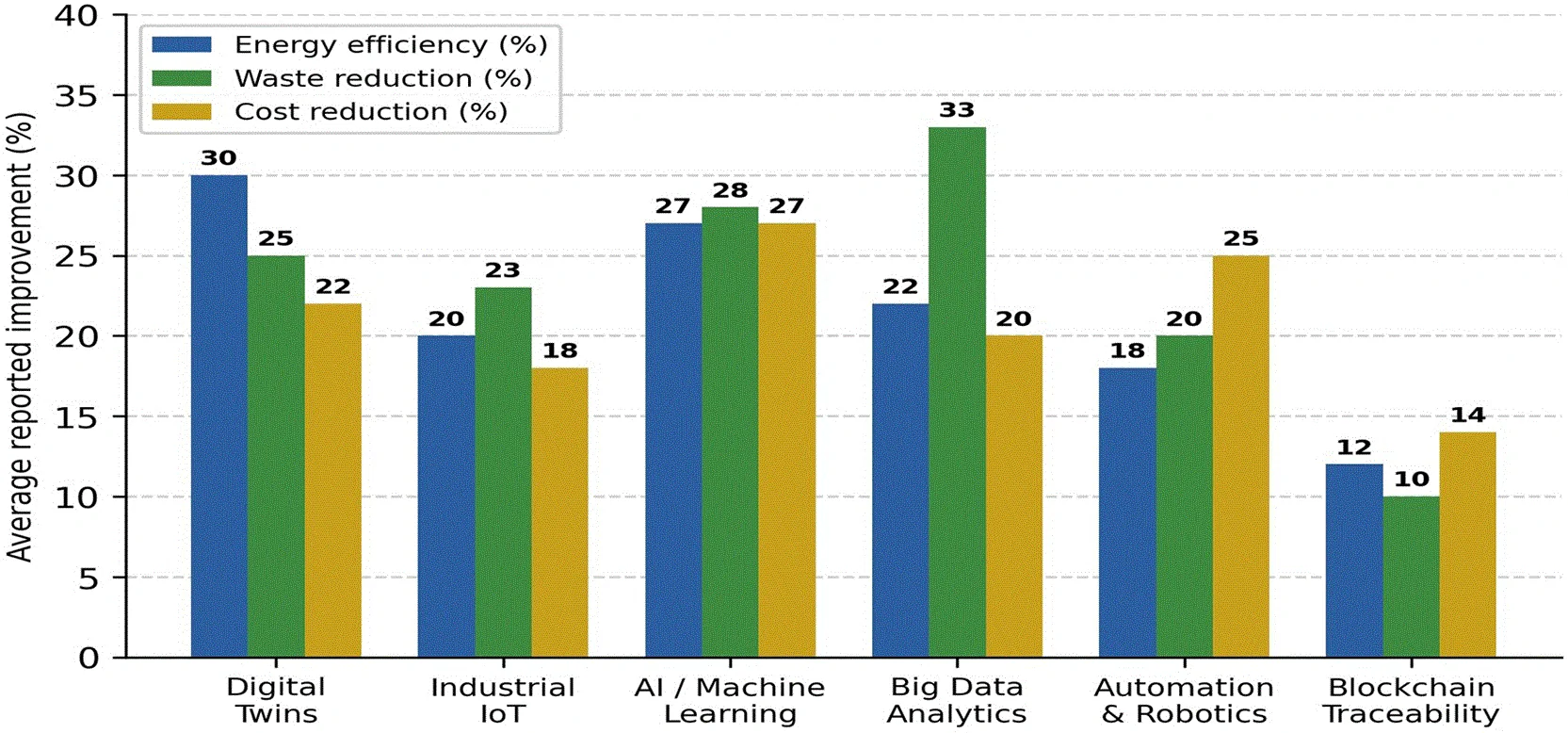
Comparative effectiveness of digital technologies in operational sustainability. The chart presents the mean improvement ranges reported in the included studies for each technology category (digital twins, industrial IoT, AI, blockchain, additive manufacturing), across three sustainability dimensions: energy efficiency, waste reduction, and operating cost reduction.

Table 2 summarises the technologies studied, their frequency, and the ranges of improvement reported in the primary studies. Digital twins were the most frequently analysed technology (22% of studies) and showed the greatest effects on process optimisation: reductions of between 25% and 35% in energy consumption and between 20% and 30% in material use, according to Singh et al. (2024) and Attaran et al. (2024). Industrial IoT (20%) facilitated continuous monitoring, with improvements of between 15% and 25% in resource utilisation. AI systems (18%) excelled in predictive planning, with reductions in operating costs of between 22% and 32% according to Parida et al. (2024). Big data analytics (16%) enabled the detection of hidden patterns of inefficiency, generating energy savings of between 18% and 26% ( Kamble et al., 2020). Figure 5 illustrates the comparative effectiveness of these technologies across sustainability indicators.

**Table 2.** Effectiveness of digital technologies in operational sustainability indicators.

Technology	Studies (%)	Energy efficiency	Waste reduction	Cost reduction
Digital twins	22	25–35	20–30	18–25%
Industrial IoT	20	15–25	18–28	15–22
AI/Machine learning	18	20–30	22–32	22–32
Big data analytics	16	18–26	25–40	16–24
Automation/robotics	14	12–20	15–25	20–30
Blockchain traceability	10	8–15	8–12	10–18

### Comparison between traditional, individual and integrated approaches

**Figure 6.**
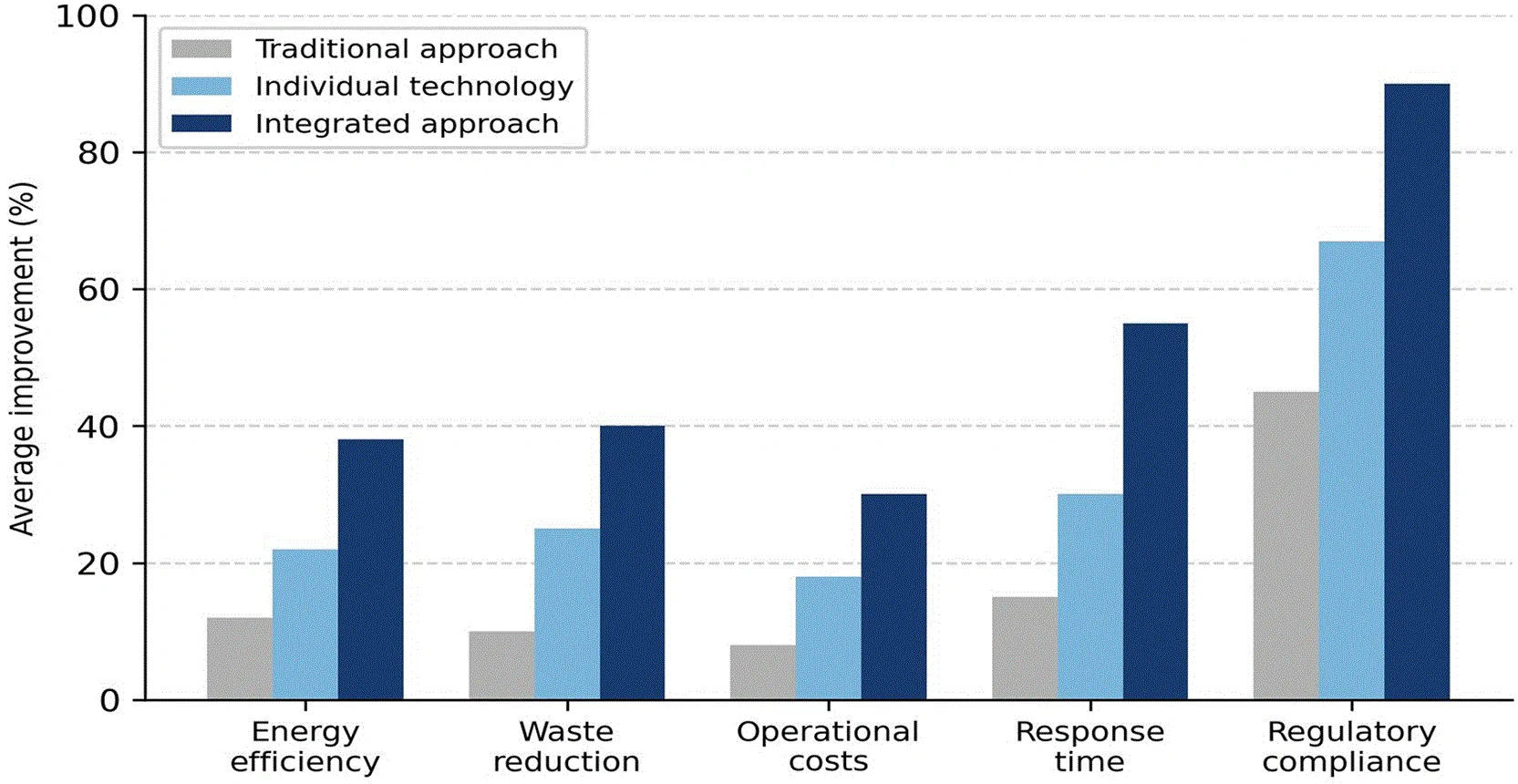
Comparison of improvements by type of approach: traditional, partially digital, and fully integrated. The figure illustrates that organisations adopting fully integrated digital approaches achieved sustainability improvements 40%–60% greater than those with traditional or fragmented implementations.

The most relevant contrast that emerges from the review is the difference in performance between three strategies: the traditional approach (reactive management based on historical data), the implementation of a single digital technology, and the integrated approach that combines three or more complementary technologies. Comparative studies indicate that organisations with integrated approaches achieved improvements of between 30% and 45% in energy efficiency, compared to between 8% and 15% with traditional approaches. Similarly, recovery times from operational disruptions were between 40% and 60% shorter in digitally mature organisations. Figure 6 illustrates this comparison.

One additional piece of data deserves attention: 78% of organisations classified as digitally mature reported successful circular economy initiatives (reuse or recycling), compared to 34% of those with traditional approaches.

### Impact on organisational practice

The aggregate results show improvements in four dimensions. In energy efficiency, studies report reductions of between 20% and 35%, with peaks of up to 45% when digital twins were combined with IoT. In material optimisation, waste reductions ranged from 25% to 40%, with improvements in inventory utilisation of between 30% and 50%. In environmental indicators, greenhouse gas emissions decreased between 22% and 38%, and industrial waste generation between 28% and 45%. Regarding regulatory compliance, 89% of the organisations studied reported improvements after implementing digital traceability systems. Table 3 summarises the contribution of the identified digital technologies to specific Sustainable Development Goals.

**Table 3.** Contribution of digital transformation to the Sustainable Development Goals.

SDG	Identified contribution	Key technologies	Evidence
SDG 9: Industry and innovation	Resilient infrastructure, sustainable industrialisation	Digital twins, IoT, AI	Ghobakhloo et al. (2025); Hasan Emon and Khan (2025)
SDG 12: Responsible production	Circular economy, life cycle traceability	Blockchain, data analytics	Karadayi-Usta (2024); Monroy-Osorio (2024)
SDG 13: Climate action	GHG emissions reduction in manufacturing	IoT, digital twins	Dong et al. (2025); Song et al. (2022)

### Sectoral differences

Effectiveness varied across sectors. The automotive and electronics industries achieved the greatest relative benefits, probably due to their greater technological maturity. The food industry showed notable progress in waste reduction and cold chain optimisation. The textile sector stood out in water and energy efficiency. Geographically, Europe and Asia-Pacific reported more mature implementations, although organisations in Latin America and Africa that achieved successful implementations showed proportionally higher rates of improvement, pointing to opportunities for technological acceleration in emerging economies ( Sepp et al., 2024).

## V. Discussion

### Summary of key findings

The results of this review confirm that combining several digital technologies produces greater sustainability benefits than each technology alone. This pattern is consistent with dynamic capabilities theory ( Xi et al., 2024) and with previous evidence from Chiarini and Kumar (2021) on the synergies between lean approaches and digital tools. The prominence of digital twins as the technology with the greatest impact validates the predictions of Singh et al. (2024) regarding their role in supply chain resilience. Framed through dynamic capabilities theory, the technologies with the largest effects in this review—digital twins, IoT and AI—map onto the sensing (real-time monitoring), seizing (scenario simulation and predictive planning) and transforming (production reconfiguration) capacities described by Xi et al. (2024); the observed advantage of integrated over isolated implementations is consistent with the idea that these three capacities are complementary rather than substitutable, so that investing in only one of them yields a partial capability.

One finding that deserves special attention is the difference between breadth and depth of digitalisation. Dong et al. (2025) found that breadth—understood as the number of digitised processes—had a greater impact on environmental resilience than the depth of implementation of a single technology. Our results reinforce this observation: organisations with integrated approaches (three or more technologies) consistently outperformed those that invested in a single tool, however sophisticated it may have been.

### Comparison with previous studies

The reported improvements for IoT (15%–25% in resource utilisation) are consistent with the findings of Luthra et al. (2020), who identified this technology as a key enabler of sustainability in supply chains. However, our ranges are somewhat more conservative than those of previous studies, which may reflect the maturation of the field and the inclusion of more recent and rigorous empirical data.

The case of blockchain is illustrative. While the initial narrative attributed transformative potential to it, the aggregate data show modest effects (8%–15% in energy efficiency). This suggests that the technology needs a more developed ecosystem—including interoperability standards and a critical mass of participants—to achieve the promised benefits.

Beyond IoT and blockchain, our finding that big data analytics generated more consistent waste-reduction benefits (25%–40%) than energy-efficiency benefits (18%–26%) is broadly consistent with Kamble et al. (2020), who emphasised the role of data-driven approaches in identifying material and process inefficiencies rather than in directly reducing energy draw. Similarly, the comparatively modest gains attributed to automation and robotics in this review align with Zekhnini et al. (2022), who argued that automation delivers sustainability benefits mainly when paired with lean process redesign rather than as a stand-alone intervention.

### Barriers limiting the digitalisation–sustainability connection

Although identifying enabling mechanisms was the primary aim of this review, several recurring barriers that constrain the digitalisation–sustainability connection also emerged from the included studies. Resource constraints limit SMEs, which represented 38% of the sample, to narrower and slower technology adoption than large multinationals. Ecosystem immaturity limits the returns from blockchain traceability, which requires interoperability standards and a critical mass of participating firms that many supply chains have not yet reached. Organisational and human factors—including workforce digital-skills gaps and resistance to process redesign—were mentioned qualitatively across several studies as moderators of implementation success, although they were rarely measured directly. Making these barriers explicit, rather than treating them only as directions for future research, helps to explain why the benefits of digital transformation are unevenly realised even among organisations investing in comparable technologies.

### Knowledge gaps and limitations

These results substantiate the scientific gap identified in the Introduction, while also revealing further limitations specific to the evidence base assembled here. This review identified three main gaps. First, the scarcity of longitudinal studies: most of the evidence comes from cross-sectional studies that do not capture the evolution of benefits over time. Second, uneven geographical representation: Europe and Asia-Pacific account for 60% of the studies, while Latin America accounts for only 12% and Africa for 4% (16% combined), consistent with the sample profile reported above. Third, the absence of standardised evaluation frameworks that allow for direct comparison of results across sectors and regions.

The limitations of the study itself must be noted transparently. The restriction to three databases may have excluded relevant studies published in sources not indexed in Scopus, ScienceDirect, or Taylor & Francis. The decision to conduct a narrative synthesis rather than a meta-analysis is due to the heterogeneity of designs and metrics, but limits the quantitative accuracy of the conclusions. Likewise, the inclusion of articles from 2025 that are not yet fully indexed introduces some uncertainty about the comprehensiveness of the sample for that year.

### Emerging challenges and future lines of research

The findings suggest at least four lines of future work. First, longitudinal designs that document the evolution of sustainability benefits over three or more years following digital implementation. Second, comparative studies between geographical contexts with different levels of development, including middle- and low-income economies. Third, interdisciplinary research incorporating perspectives from organisational psychology and sociology of work to understand human barriers to technology adoption. Fourth, exploration of emerging technologies—such as generative artificial intelligence and quantum computing—and their potential impact on manufacturing sustainability.

### Implications for practice

For manufacturing executives, the main operational conclusion is that investments in digitalisation for sustainability generate both environmental and economic returns, with operating cost reductions of between 18% and 32%. However, these benefits materialise more strongly when implementation is integrated rather than fragmented. SMEs, with more limited resources, can start with low-complexity IoT systems before scaling up to more sophisticated solutions.

For public policymakers, the results underscore the importance of designing regulatory frameworks and incentives that facilitate technology adoption, particularly in emerging economies where infrastructure and training gaps are more pronounced.

## VI. Conclusions

This systematic review of 50 studies published between 2015 and 2025 yields three main conclusions. First, the joint implementation of digital technologies—especially digital twins, IoT, and artificial intelligence—generates measurable improvements in energy efficiency (20%–35%), waste reduction (25%–40%), and operating costs (18%–32%). Second, integrated approaches consistently outperform isolated implementations, with gains between 40% and 60% higher in sustainability indicators. Third, significant gaps remain: longitudinal studies are lacking, geographical representation is uneven, and standardised evaluation frameworks do not exist.

The study contributes to knowledge by offering a structured synthesis of the mechanisms that connect digitalisation with operational sustainability and by explicitly mapping the gaps that future research should address. For business practice, the central message is that digitalisation and sustainability are not parallel agendas but mutually reinforcing when designed in an integrated manner.

The future research agenda should prioritise longitudinal designs, comparative studies between regions with different levels of development, interdisciplinary research on human and organisational barriers, and the evaluation of emerging technologies such as generative artificial intelligence. Only through this expansion of knowledge will it be possible to more accurately guide the transition to genuinely sustainable manufacturing in the digital age.

## Data Availability

This article is a systematic review. All data supporting the results reported in the article are available within the article itself and its supplementary materials. The list of included studies and the data extraction tables are available as Extended Data files accompanying this submission. The underlying datasets from the primary studies reviewed are available in their respective original publications, all of which are cited in the reference list. No new primary datasets were generated for this study. The PRISMA 2020 checklist for this review has been uploaded as a supplementary file and is available at: https://doi.org/10.6084/m9.figshare.31585258 ( Cruz Salinas, et al., 2025a). This dataset is available under a CC-BY 4.0 licence. Extended Data Table 1. Full data extraction table for the 50 included studies, including study design, digital technologies analysed, sustainability outcomes reported, and methodological quality scores. Available at: https://doi.org/10.6084/m9.figshare.31585795 ( Cruz Salinas, et al., 2025b). This dataset is available under a CC-BY 4.0 licence .
