# Supplementary material for: Digital transformation as a driver of operational sustainability in manufacturing: a systematic review (2015–2025)
Source: F1000Res. 2026 Sep 16;15:544. Originally published 2026 Apr 16. [Version 2] doi: 10.12688/f1000research.178740.2 (PMC13146463; doi:10.12688/f1000research.178740.2)
Supplement: Peer review history

# Reviewer report: Reviewer response for version 2

**DOI:** 10.5256/f1000research.200737.r521958

**Published:** 2026-09-18

**Authors:** Benny Hutahayan

**Affiliations:** 1 University of Brawijaya, Malang, East Java, Indonesia

The author has made the revisions properly, in accordance with my suggestions.

Are the rationale for, and objectives of, the Systematic Review clearly stated?

Yes

Is the statistical analysis and its interpretation appropriate?

Partly

If this is a Living Systematic Review, is the ‘living’ method appropriate and is the search schedule clearly defined and justified? (‘Living Systematic Review’ or a variation of this term should be included in the title.)

Not applicable

Are sufficient details of the methods and analysis provided to allow replication by others?

Partly

Are the conclusions drawn adequately supported by the results presented in the review?

Yes

Reviewer Expertise:

Business Administration, Human Resources Management, Law, and Governance

I confirm that I have read this submission and believe that I have an appropriate level of expertise to confirm that it is of an acceptable scientific standard.

# Reviewer report: Reviewer response for version 1

**DOI:** 10.5256/f1000research.197164.r475966

**Published:** 2026-06-13

**Authors:** Nur Azaliah Abu Bakar

**Affiliations:** 1 Universiti Teknologi Malaysia, Skudai, Johor, Malaysia

This systematic review examines the role of digital transformation technologies, including Industrial IoT, artificial intelligence, digital twins, big data analytics, automation, and blockchain, in improving operational sustainability within the manufacturing sector. Following PRISMA 2020 guidelines, the authors reviewed literature published between 2015 and 2025 and synthesized evidence from 50 studies selected from an initial pool of 1,247 records. The review aims to identify the technologies with the greatest sustainability impact, compare isolated versus integrated digital implementations, and identify knowledge gaps for future research.

 The rationale and objectives of the review are clearly stated. The manuscript establishes the importance of sustainable manufacturing and the growing role of Industry 4.0 technologies in addressing environmental and operational challenges. The research questions and review objectives are explicitly defined and aligned with the scope of the study.

 The methodology generally follows accepted systematic review practices and includes database selection, search terms, inclusion and exclusion criteria, quality assessment procedures, and a PRISMA flow diagram. However, the level of methodological detail remains insufficient for full replication. The manuscript does not provide the exact search strings used in each database, the search dates, database-specific adaptations, detailed quality assessment scoring results, or inter-rater agreement statistics. Although a quality appraisal process is described, the operationalisation of the adapted Downs and Black and Tracy criteria is not reported in sufficient detail. These elements should be added to enhance transparency and reproducibility.

 The statistical analysis is only partly satisfactory. The study appropriately employs a narrative synthesis because of the heterogeneity of study designs and outcome measures. However, the manuscript reports numerous quantitative improvement ranges, percentages, and comparative performance figures without fully explaining how these values were aggregated across studies. Since no formal meta-analysis was conducted, the basis for calculating average improvements and comparative effectiveness should be clarified. The authors should explicitly state whether these values represent reported ranges from primary studies, weighted averages, or descriptive summaries derived during synthesis.

 The conclusions are generally supported by the evidence presented. The review consistently demonstrates that integrated digital transformation initiatives outperform isolated technology implementations and are associated with improvements in energy efficiency, waste reduction, operational performance, and sustainability outcomes. The discussion appropriately acknowledges limitations related to geographical representation, lack of longitudinal evidence, database coverage, and methodological heterogeneity. These limitations provide an appropriate context for interpreting the findings.

 To strengthen the scientific rigor of the review, the authors should address several points. First, provide complete and reproducible search protocols, including database-specific search strings and search dates. Second, report detailed methodological quality assessment results for all included studies. Third, clarify how quantitative improvement ranges and comparative percentages were synthesized across the included studies. Fourth, provide a more detailed explanation of how the narrative and thematic synthesis procedures were conducted. Finally, several numerical claims should be presented more cautiously because they are derived from heterogeneous studies and should not be interpreted as pooled effect estimates.

 Overall, the review addresses an important and timely topic, presents a coherent synthesis of the literature, and offers useful implications for manufacturing sustainability. Subject to improvements in methodological transparency and clarification of the quantitative synthesis procedures, the article provides a valuable contribution to the field of digital transformation and sustainable manufacturing.

Are the rationale for, and objectives of, the Systematic Review clearly stated?

Yes

Is the statistical analysis and its interpretation appropriate?

Partly

If this is a Living Systematic Review, is the ‘living’ method appropriate and is the search schedule clearly defined and justified? (‘Living Systematic Review’ or a variation of this term should be included in the title.)

Yes

Are sufficient details of the methods and analysis provided to allow replication by others?

Yes

Are the conclusions drawn adequately supported by the results presented in the review?

Partly

Reviewer Expertise:

Artificial Intelligence, Data Analytics, Enterprise Architecture, Digital Transformation, Information Systems Governance, Educational Technology, Cybersecurity, and Higher Education Internationalisation.

I confirm that I have read this submission and believe that I have an appropriate level of expertise to confirm that it is of an acceptable scientific standard.

# Reviewer report: Reviewer response for version 1

**DOI:** 10.5256/f1000research.197164.r475971

**Published:** 2026-05-05

**Authors:** Sandra Lizzette León Luyo

**Affiliations:** 1 Universidad Nacional de Trujillo, Trujillo, Peru

When conducting a study based on a systematic review of information from 2015 to 2025, following the PRISMA 2020 protocol, on topics related to core Industry 4.0 technologies —such as industrial IoT, artificial intelligence, digital twins, and big data analytics— it becomes possible to visualize how these technologies influence the sustainable performance of a sector sustained by global GDP figures. Likewise, the findings are precise and applicable, indicating that the use of these technologies promotes a reduction in energy consumption of between 20% and 35%, and that the generation of material waste decreases between 25% and 40%; these figures are particularly relevant for business decision-makers as well as for those responsible for developing public policies aimed at meeting SDGs 9 and 12. On the other hand, the study highlights the scarcity of longitudinal studies and the low participation of research from Latin America and Africa, giving rise to the need to close academic gaps, for which reason a more inclusive and long-term research agenda is recommended.

Are the rationale for, and objectives of, the Systematic Review clearly stated?

Yes

Is the statistical analysis and its interpretation appropriate?

Yes

If this is a Living Systematic Review, is the ‘living’ method appropriate and is the search schedule clearly defined and justified? (‘Living Systematic Review’ or a variation of this term should be included in the title.)

No

Are sufficient details of the methods and analysis provided to allow replication by others?

Yes

Are the conclusions drawn adequately supported by the results presented in the review?

Yes

Reviewer Expertise:

Environmental culture, finance, entrepreneurship, international trade

I confirm that I have read this submission and believe that I have an appropriate level of expertise to confirm that it is of an acceptable scientific standard.

# Reviewer report: Reviewer response for version 1

**DOI:** 10.5256/f1000research.197164.r479087

**Published:** 2026-04-30

**Authors:** Heyner Yuliano Marquez Yauri

**Affiliations:** 1 Universidad Nacional de Trujillo, Trujillo, Peru

This article analyzes how digital transformation drives sustainability in manufacturing through a systematic literature review (2015-2025). The study is relevant given the current need for companies to be both competitive and sustainable. Despite the inherent limitations of heterogeneous research designs and the geographical diversity of the analyzed studies, this work offers a key combination of insights into how digital technologies optimize production processes and reduce environmental impact.

 Methodologically, the article demonstrates solid methodological rigor by adhering to the PRISMA 2020 guidelines, a gold standard for systematic reviews.

 While the study contributes to knowledge by offering a structured synthesis of the mechanisms connecting digitalization with operational sustainability, it overlooks or fails to address the mechanisms or barriers that limit this connection (critical challenges). Although not the study's objective, this is an aspect that should be clarified or addressed.

Are the rationale for, and objectives of, the Systematic Review clearly stated?

Yes

Is the statistical analysis and its interpretation appropriate?

Yes

If this is a Living Systematic Review, is the ‘living’ method appropriate and is the search schedule clearly defined and justified? (‘Living Systematic Review’ or a variation of this term should be included in the title.)

No

Are sufficient details of the methods and analysis provided to allow replication by others?

Yes

Are the conclusions drawn adequately supported by the results presented in the review?

Yes

Reviewer Expertise:

Business strategiesDigital transformation and marketingInternational tradeSustainability and environmental cultureEntrepreneurship and innovationEducational management

I confirm that I have read this submission and believe that I have an appropriate level of expertise to confirm that it is of an acceptable scientific standard.

# Reviewer report: Reviewer response for version 1

**DOI:** 10.5256/f1000research.197164.r479088

**Published:** 2026-04-29

**Authors:** Benny Hutahayan

**Affiliations:** 1 University of Brawijaya, Malang, East Java, Indonesia

Introduction

 - The research gap needs to be further clarified. Currently, the gap is primarily presented as a list of general limitations, such as the lack of longitudinal studies and geographical representation, but has not yet been fully articulated as a scientific gap that directly justifies the need for this systematic review.

 - The novelty of the research has not been explicitly stated. The authors need to clarify what distinguishes this review from previous review studies, for example, in terms of the time period covered, the focus on operational sustainability, or the comparison between integrated and partial implementation.  

 - The 2015–2025 timeframe has been mentioned, but no sufficiently strong justification has been provided. The authors need to explain why this period was chosen and its relevance to the development of Industry 4.0 in manufacturing.

 Methodology

 - The search strategy needs to be described in greater detail to ensure it can be replicated, including the search date, search fields (title, abstract, keywords), and whether the search syntax was tailored for each database.

 - The inclusion and exclusion criteria are mentioned in the manuscript, but the authors need to provide a justification for why and on what basis these criteria were established. This explanation is necessary to strengthen the manuscript.

 - The quality assessment section demonstrates efforts to maintain methodological rigor through two independent reviewers, but the assessment procedures still need to be described in more operational terms. The authors need to explain in greater detail how the instruments from Downs and Black (1998) and Tracy (2010) were adapted, which items were used, and how the quality scores were calculated. Therefore, the authors need to provide the results of the quality assessment in detail.

**- **The discussion section has begun to link the review’s findings to theory and prior research, particularly dynamic capabilities theory and previous studies on the synergy between lean approaches and digital tools. Comparisons with prior studies are present but could be expanded to be more systematic. The discussion of the relationship between the findings and dynamic capabilities theory remains relatively brief. If this theory is indeed the primary foundation, then the author needs to demonstrate this more explicitly.

Are the rationale for, and objectives of, the Systematic Review clearly stated?

Yes

Is the statistical analysis and its interpretation appropriate?

Partly

If this is a Living Systematic Review, is the ‘living’ method appropriate and is the search schedule clearly defined and justified? (‘Living Systematic Review’ or a variation of this term should be included in the title.)

Not applicable

Are sufficient details of the methods and analysis provided to allow replication by others?

Partly

Are the conclusions drawn adequately supported by the results presented in the review?

Yes

Reviewer Expertise:

Business Administration, Human Resources Management, Law, and Governance

I confirm that I have read this submission and believe that I have an appropriate level of expertise to confirm that it is of an acceptable scientific standard, however I have significant reservations, as outlined above.

# Response

**Published:** 2026-04-30

**Authors:** Luis Cruz Salinas

**Affiliations:** Ingeniería Industrial, Universidad Cesar Vallejo, Trujillo, La Libertad, Peru

**Comment R2.1 — Introduction. Research gap.**

**Reviewer comment.**

*The research gap needs to be further clarified. Currently, the gap is primarily presented as a list of general limitations, such as the lack of longitudinal studies and geographical representation, but has not yet been fully articulated as a scientific gap that directly justifies the need for this systematic review.*

**Author response.**

 We agree. The previous wording named field-level limitations rather than a specific scientific gap that requires a new review. The closing paragraph of Section 1 has been rewritten so the gap is stated in terms of what previous syntheses have not done and why that omission matters for practice and policy.

**Manuscript change.**

 Location: Section 1 (Introduction), end of the last paragraph. The exact wording inserted (visible as a tracked change in the revised file) is:

*“To make the gap explicit: although the literature documents partial effects of individual technologies on environmental indicators, no review has yet integrated the evidence on how the combined adoption of IoT, AI, digital twins and big data shapes operational sustainability outcomes during the 2015–2025 window. This absence prevents practitioners and policymakers from knowing which combinations of technologies produce measurable gains, under which conditions, and with what magnitude. The present review addresses that specific gap by mapping technologies to operational sustainability indicators and contrasting integrated versus single-technology implementations.”*

**Comment R2.2 — Introduction. Novelty.**

**Reviewer comment.**

*The novelty of the research has not been explicitly stated. The authors need to clarify what distinguishes this review from previous review studies, for example, in terms of the time period covered, the focus on operational sustainability, or the comparison between integrated and partial implementation.*

**Author response.**

 We have added a paragraph in Section 1 stating the three specific differences between our review and earlier syntheses: the time window (2015–2025), the unit of analysis (operational sustainability rather than environmental performance in the abstract), and the explicit comparison of integrated versus single-technology implementations.

**Manuscript change.**

 Location: Section 1 (Introduction), new paragraph after the literature trajectory. The exact wording inserted (visible as a tracked change in the revised file) is:

*“Three features distinguish this review from earlier syntheses. First, the 2015–2025 window covers the full diffusion cycle of Industry 4.0 in manufacturing, from early pilots to large-scale rollouts, which earlier reviews ending in 2021 or 2022 could not capture. Second, the unit of analysis is operational sustainability, meaning energy use, material waste and downtime, rather than environmental performance in the abstract; this is what manufacturing decision-makers actually measure and act on. Third, the review systematically contrasts integrated implementations (three or more digital technologies adopted jointly) with partial or single-technology implementations, a comparison that has not been made directly in previous reviews.”*

**Comment R2.3 — Introduction. Timeframe justification.**

**Reviewer comment.**

*The 2015–2025 timeframe has been mentioned, but no sufficiently strong justification has been provided. The authors need to explain why this period was chosen and its relevance to the development of Industry 4.0 in manufacturing.*

**Author response.**

 Justification has been added to Section 1, anchoring the lower bound to the 2015 consolidation of Industry 4.0 as a manufacturing programme and the upper bound to the first decade of measured outcomes, including the post-pandemic acceleration of digital investment.

**Manuscript change.**

 Location: Section 1 (Introduction), inserted after the research question. The exact wording inserted (visible as a tracked change in the revised file) is:

*“This window was chosen for two reasons. The starting point of 2015 marks the year in which Industry 4.0 moved from conceptual discussion to documented industrial deployment, particularly after the publication of Germany's Plattform Industrie 4.0 implementation roadmap and the parallel emergence of standardised IoT protocols. The closing point of 2025 captures the first decade of measured outcomes, including the post-pandemic acceleration of digital investment in manufacturing reported by Dong et al. (2025) and Attaran et al. (2024).”*

**Comment R2.4 — Methodology. Search strategy.**

**Reviewer comment.**

*The search strategy needs to be described in greater detail to ensure it can be replicated, including the search date, search fields (title, abstract, keywords), and whether the search syntax was tailored for each database.*

**Author response.**

 The Methodology has been expanded to include the actual dates of the searches, the fields targeted in each database, and the per-database adaptations of the Boolean string. Search logs are available on request.

**Manuscript change.**

 Location: Section 3 (Methodology), Search strategy subsection. The exact wording inserted (visible as a tracked change in the revised file) is:

*“Searches were executed between 12 and 26 March 2025 by two reviewers working in parallel. In each database the query was applied to the title, abstract and author-keyword fields (TITLE-ABS-KEY in Scopus, the equivalent fielded search in ScienceDirect, and the All Fields except Full Text option in Taylor & Francis Online). The Boolean string was adapted to the syntactic requirements of each platform: Scopus accepted the full nested structure; ScienceDirect required the operators to be split into two paired queries because of its eight-Boolean limit; Taylor & Francis Online required the replacement of curly quotation marks with straight ones and the use of NEAR/3 instead of AND for the digitalisation–sustainability pairing. Search logs were exported and are available on request.”*

**Comment R2.5 — Methodology. Inclusion and exclusion criteria.**

**Reviewer comment.**

*The inclusion and exclusion criteria are mentioned in the manuscript, but the authors need to provide a justification for why and on what basis these criteria were established. This explanation is necessary to strengthen the manuscript.*

**Author response.**

 A new paragraph after Table 1 explains the basis for each criterion: the temporal cut-off, the restriction to peer-reviewed journals, the manufacturing-only scope and the language criterion.

**Manuscript change.**

 Location: Section 3 (Methodology), new paragraph after Table 1. The exact wording inserted (visible as a tracked change in the revised file) is:

*“The criteria were set on four grounds. The temporal cut-off of 2015 corresponds to the consolidation of Industry 4.0 as a manufacturing programme, as discussed in Section 1; earlier work belongs to a different technological generation. The restriction to peer-reviewed journal articles was adopted to ensure that each included study had passed an external quality filter, given that grey literature on digital transformation tends to be promotional. The manufacturing-only scope reflects the review question, since service or agriculture settings have different operational sustainability indicators and would dilute the synthesis. The English-and-Spanish language criterion was set because these two languages cover the bulk of indexed manufacturing research and align with the reviewers' working languages, so screening could be done without translation-induced misclassification.”*

**Comment R2.6 — Methodology. Quality assessment.**

**Reviewer comment.**

*The quality assessment section demonstrates efforts to maintain methodological rigor through two independent reviewers, but the assessment procedures still need to be described in more operational terms. The authors need to explain in greater detail how the instruments from Downs and Black (1998) and Tracy (2010) were adapted, which items were used, and how the quality scores were calculated. Therefore, the authors need to provide the results of the quality assessment in detail.*

**Author response.**

 The quality assessment subsection has been expanded with the operational detail requested: the specific items retained from Downs and Black (1998), the rubric used to score the eight Tracy (2010) markers, the rescaling that allows quantitative and qualitative streams to be compared, the inter-reviewer reconciliation rule and the actual distribution of quality scores across the 50 included studies. The full per-study scoresheet is provided as supplementary material S2.

**Manuscript change.**

 Location: Section 3 (Methodology), Methodological quality assessment subsection. The exact wording inserted (visible as a tracked change in the revised file) is:

*“From the original 27-item Downs and Black checklist, items 1–10 (reporting), items 11–13 (external validity) and items 14–20 (internal validity, excluding the items specific to randomised trials) were retained, giving a 20-item subset for quantitative studies. Each item scored 0 or 1, except items 5 and 27 which were dichotomised against the original 0–2 scale. For qualitative work, the eight Tracy markers (worthy topic, rich rigour, sincerity, credibility, resonance, significant contribution, ethics and meaningful coherence) were each scored on a 0–2 rubric and the sum rescaled to the 0–10 scale used for the quantitative checklist, so the two streams could be compared. Discrepancies above one point between the two reviewers were resolved by a third evaluator. Of the 50 included studies, 31 reached the high-quality threshold (≥7), 17 fell in the moderate range (5–6) and 2 in the low range; the two low-quality studies were retained because they reported primary data not available elsewhere, and their findings are flagged in the synthesis. The full per-study scoresheet is provided as supplementary material S2.”*

**Comment R2.7 — Discussion. Link to dynamic capabilities theory.**

**Reviewer comment.**

*The discussion section has begun to link the review's findings to theory and prior research, particularly dynamic capabilities theory and previous studies on the synergy between lean approaches and digital tools. Comparisons with prior studies are present but could be expanded to be more systematic. The discussion of the relationship between the findings and dynamic capabilities theory remains relatively brief. If this theory is indeed the primary foundation, then the author needs to demonstrate this more explicitly.*

**Author response.**

 We have added a paragraph in the Discussion that maps the empirical findings onto the three dynamic capability dimensions (sensing, seizing, reconfiguring) and shows that the empirical pattern follows the order the theory predicts.

**Manuscript change.**

 Location: Section 5 (Discussion), Summary of key findings subsection. The exact wording inserted (visible as a tracked change in the revised file) is:

*“Mapping the findings onto the three dynamic capability dimensions makes the connection more explicit. The sensing capacity of Teece (2007) corresponds to the IoT and big-data layer in our review: the firms that achieved the largest energy and waste reductions were those whose sensing infrastructure produced continuous, plant-wide signals rather than periodic snapshots. The seizing capacity corresponds to the analytics and digital-twin layer, where firms turned the sensed data into operational decisions, including production scheduling and predictive maintenance; the 25–40% material-waste reduction reported in the integrated cases is exactly what one would expect when seizing is well-instrumented. The reconfiguring capacity corresponds to the organisational redesign that accompanied integrated implementations: firms that adopted three or more technologies jointly also reorganised maintenance, quality and energy-management functions, which is consistent with the 40–60% relative gain we observed for integrated versus single-technology adopters. The empirical pattern is therefore consistent with dynamic capabilities theory, and it follows the order the theory predicts: reconfiguring produces the largest marginal benefit, in line with Eisenhardt and Martin (2000).”*
